# Evaluation of pulse pressure variation with different inhaled concentrations of desfluorane, sevofluorane and isofluorane in pigs

**DOI:** 10.1186/cc9476

**Published:** 2011-03-11

**Authors:** AH Oshiro, DT Fantoni, DA Otsuki, KT Rosa, JO Auler

**Affiliations:** 1 Faculdade de Medicina da universidade de São Paulo, Brazil

## Introduction

Pulse pressure variation (PPV) has been shown to predict preload fluid responsiveness in mechanically ventilated patients [1]. Inhalant anesthetic agents have dose-dependent hemodynamic and direct myocardial contractility effects. The aim of this study was to compare the behavior of PPV under desfluorane, sevofluorane and isofluorane anesthesia.

## Methods

Twenty-four anesthetized and mechanically ventilated pigs were randomly assigned into three groups of eight animals: desfluorane (DESF), sevofluorane (SEVO) and isofluorane (ISO). Static hemodynamic parameters and PPV, measured by pulmonary artery and femoral arterial catheters, were assessed at baseline (T1) using 1 MAC of the volatile agent; T2 (1.25 MAC); T3 (1 MAC) and T4 (1.0 MAC associated with a 30% hemorrhage of estimated average volemia). Two-way ANOVA and Tukey test were used for statistical analysis (*P *< 0.05).

## Results

At T2 there was an increase in PPV in all groups but not statistically significant compared with T1 or among groups. At T4 the increase in PPV was significant compared with basal values in the three groups: DESF (11 ± 4 vs. 7 ± 2%, *P *< 0.001); SEVO (15 ± 5 vs. 6 ± 2%, *P *< 0.001) and ISO (14 ± 5 vs. 7 ± 3%, *P *< 0.001). No statistical difference between groups was found for PPV. Mean arterial pressure (MAP) decreased after 25% increment of MAC (T2) and after hemorrhage. At T4, MAP decreased significantly lower than basal values (T1) in groups DESF (*P *< 0.001), SEVO (*P *< 0.001) and ISO (*P *< 0.001). Cardiac index (CI) decreased in T2 compared with T1: DESF (3.6 ± 0.6 vs. 2.9 ± 0.5 l/min/m^2^, *P *< 0.001), SEVO (4.0 ± 0.1 vs. 3.1 ± 0.4 l/min/m^2^, *P *< 0.001) and ISO (4.2 ± 0.1 vs. 3.6 ± 0.9, *P *< 0.001). The CI drop after hemorrhage showed no statistical difference when compared with T1.

## Conclusions

PPV behaved similarly with different inhaled anesthetics. Although PPV did not reflect the hemodynamic depression of incrementing MAC values, it increased after bleeding 30% of estimated volemia.
